# New structural and functional defects in polyphosphate deficient bacteria: A cellular and proteomic study

**DOI:** 10.1186/1471-2180-10-7

**Published:** 2010-01-12

**Authors:** Cristian Varela, Cecilia Mauriaca, Alberto Paradela, Juan P Albar, Carlos A Jerez, Francisco P Chávez

**Affiliations:** 1 Laboratory of Molecular Microbiology and Biotechnology & Millennium Institute of Cell Dynamics and Biotechnology (ICDB), Department of Biology, Faculty of Sciences, University of Chile, Las Palmeras 3425, Ñuñoa, Santiago, Chile; 2 Servicio de Proteómica, Centro Nacional de Biotecnología. CSIC. Darwin 3, 28049, Madrid, España

## Abstract

**Background:**

Inorganic polyphosphate (polyP), a polymer of tens or hundreds of phosphate residues linked by ATP-like bonds, is found in all organisms and performs a wide variety of functions. PolyP is synthesized in bacterial cells by the actions of polyphosphate kinases (PPK1 and PPK2) and degraded by exopolyphosphatase (PPX). Bacterial cells with polyP deficiencies due to knocking out the *ppk1 *gene are affected in many structural and important cellular functions such as motility, quorum sensing, biofilm formation and virulence among others. The cause of this pleiotropy is not entirely understood.

**Results:**

The overexpression of exopolyphosphatase in bacteria mimicked some pleitropic defects found in *ppk1 *mutants. By using this approach we found new structural and functional defects in the polyP-accumulating bacteria *Pseudomonas sp*. B4, which are most likely due to differences in the polyP-removal strategy. Colony morphology phenotype, lipopolysaccharide (LPS) structure changes and cellular division malfunction were observed. Finally, we used comparative proteomics in order to elucidate the cellular adjustments that occurred during polyP deficiency in this bacterium and found some clues that helped to understand the structural and functional defects observed.

**Conclusions:**

The results obtained suggest that during polyP deficiency energy metabolism and particularly nucleoside triphosphate (NTP) formation were affected and that bacterial cells overcame this problem by increasing the flux of energy-generating metabolic pathways such as tricarboxilic acid (TCA) cycle, β-oxidation and oxidative phosphorylation and by reducing energy-consuming ones such as active transporters and amino acid biosynthesis. Furthermore, our results suggest that a general stress response also took place in the cell during polyP deficiency.

## Background

Polyphosphate (polyP) is a ubiquitous linear polymer of hundreds of orthophosphate residues (Pi) linked by phosphoanhydride bonds. PolyP has been found in all tree domains of life (*Archaea, Bacteria *and *Eukarya*). In bacteria, the main enzymes involved in the metabolism of polyP are the polyphosphate kinases (PPK1 and PPK2) that catalyze the reversible conversion of the terminal phosphate of ATP (or GTP) into polyP and the exopolyphosphatase (PPX) that processively hydrolyzes the terminal residues of polyP to liberate Pi [1,2].

PolyP is a reservoir of phosphate and, as in ATP, of high-energy phosphate bonds. Furthermore, biochemical experiments and studies with *ppk1 *mutants in many bacteria have indicated additional roles for polyP. These include inhibition of RNA degradation [3], activation of Lon protease during stringent response [4,5], involvement in membrane channel structure [6,7], and contribution to the resistance to stress generated by heat, oxidants, osmotic challenge, antibiotics and UV [8-12]. Particularly, a *ppk1 *mutant of *Pseudomonas aeruginosa *PAO1 was impaired in motility, biofilm development, quorum sensing and virulence [13-15].

In addition to PPK1, another widely conserved polyP enzyme is PPK2 [16,17]. In contrast to the ATP-dependent polyP synthetic activity of PPK1, PPK2 preferentially catalyses the polyP-driven synthesis of GTP from GDP. Orthologs to both proteins have been found in many bacterial genomes and curiously there are many bacteria with orthologs of either PPK1 or PPK2, or both, or neither [17].

PolyP in bacteria is localized predominantly in volutin granules, also called polyP granules, or in acidocalcisomes [18]. Many biochemical pathways are connected and a given metabolite such as polyP can be generated and/or consumed by several enzymes or cellular processes. The genetic background, culture conditions and environmental factors can influence polyP levels. Its absence, as mentioned above, causes many structural and functional defects. The link between genotypes and phenotypes observed during polyP deficiency can be the result of complex networks of interaction that can be elucidated by using OMICS technology [19,20].

Recombinant *Pseudomonas sp*. B4 that overexpressed yeast exopolyphosphatase also showed the functional deficiencies in motility and biofilm development reported for *ppk1 *mutants from *P. aeruginosa *PAO1 [21]. In addition, new structural and functional defects such as changes in colony morphology, LPS structure and cellular division are reported in this communication. Finally, to study the proteomic changes that occurred during polyP deficiency recombinant strains were compared under different growth conditions and phases of growth. Interesting proteins related to energetic metabolism were overexpressed during polyP scarcity, such as three enzymes from the tricarboxylic acid (TCA) cycle, and one ATP synthase subunit. Protein folding, fatty acid catabolism and amino acid biosynthesis were other gene onthology (GO) categories overrepresented during polyP deficit. On the other hand, motility and transport proteins were the only categories underrepresented in this condition.

The proteomics results suggest a link between polyP and central metabolism that can be further explored to clarify the multiple structural and functional defects found during the lack of polyP in bacteria.

## Results

### Structural and functional defects in polyphosphate deficient bacteria

**Figure 1:**
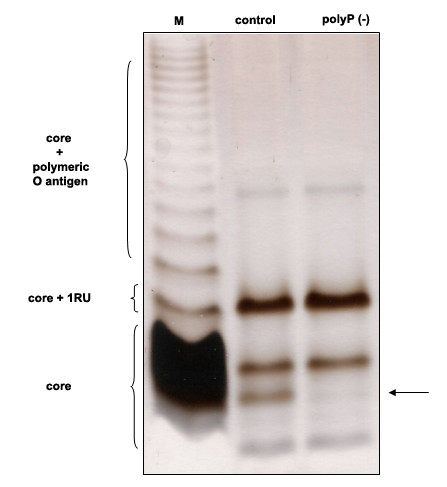
**LPS profiles of polyP-deficient cells of *Pseudomonas sp*. B4**. Equal numbers of *Pseudomonas *sp. B4 polyP-deficient and control cell samples were loaded in each lane and analysed by 12% (w/v) PAGE by using a Tricine-SDS buffer system. LPS from *Salmonella serovars *Typhi was used as LPS control (lane M). The arrow indicates the change seen in a band of the inner core. RU: repetitive units.

Overexpression of PPX resembled the functional defects found in motility and biofilm formation in a *ppk1 *mutant from *P. aeruginosa *PAO [21]. Despite several functional and structural defects have been reported in *P. aeruginosa *PAO1 *ppk1 *mutant [15,21,22], our polyP deficient cells showed new functional and structural phenotypes not previously reported. PPK1 is essential for biofilm development and virulence of *P. aeruginosa *PAO1. Considering that lipopolysaccharide (LPS) is also very important in both cellular processes; the electrophoretic profile of LPS from recombinants *Pseudomonas sp*. B4 were analyzed. Interestingly, changes in the core of the LPS were observed in Tricine/SDS-polyacrylamide gel electrophoresis (Figure 1). To our knowledge, the structure of the LPS core from *Pseudomonas sp*. B4 has not yet been elucidated and consequently it is difficult to determine the structural nature of the change found in the LPS core. It would be interesting to determine the structure of LPS in both strains [control and polyP(-)] to reveal the change in the LPS and its probable link with polyP.

**Figure 2:**
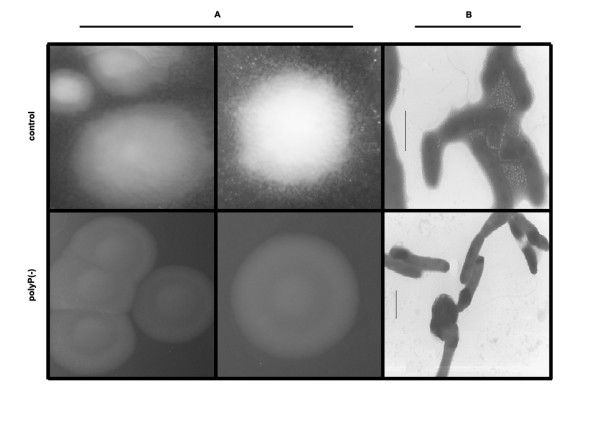
**Colony morphology of polyP-deficient cells of *Pseudomonas sp*. B4**. *Pseudomonas sp*. B4 polyP-deficient and control cells were grown in LB plates for 48 h and the colonies were photographed by using a magnifying glass (A). Unstained cells were analyzed by transmission electron microscopy (B).

It was found that inorganic polyP influences not only biofilm formation but also colony morphology phenotype. Changes were seen when the colony phenotype of control cells was compared with that of polyP-deficient ones. Wild type and control cells were highly motile forming a rough colony with an irregular border (Figure 2A). In contrast, polyP-deficient cells displayed a round regular smooth colony (Figure 2A). The change observed in colony morphology could be directly a consequence of the absence of exopolymer production observed in the cells (Figure 2B) and in a *P. aeruginosa *PAO1 *ppk1 *mutant [22] but also due to the variation in the LPS core reported here. Altogether, the results suggest that biofilm formation capabilities of polyP-deficient mutants, may not only be attributed to the defect in exopolymer formation, but also to their altered LPS structure.

**Figure 3:**
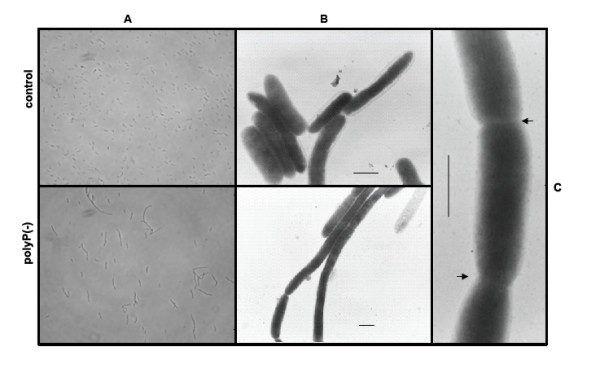
**PolyP-deficient cells become filamentous during stationary phase of growth**. *Pseudomonas sp*. B4 polyP-deficient and control cells were grown in LB medium and observed by using phase contrast-optical microscopy (A) and transmission electron microscopy of unstained cells (B). Magnified view of polyP-deficient cells (C). Arrows indicate the septum.

Finally, during the entrance in stationary phase of growth in rich medium (LB) it was observed that polyP-deficient cells became highly filamentous compared to control cells most likely reflecting a cell division malfunction (Figure 3). Different defined media supplemented with various carbon sources were tested and this behaviour was found only during the entry into the stationary phase of growth in LB medium.

### Differential proteomics of polyP-deficient *Pseudomonas sp*. B4

**Figure 4:**
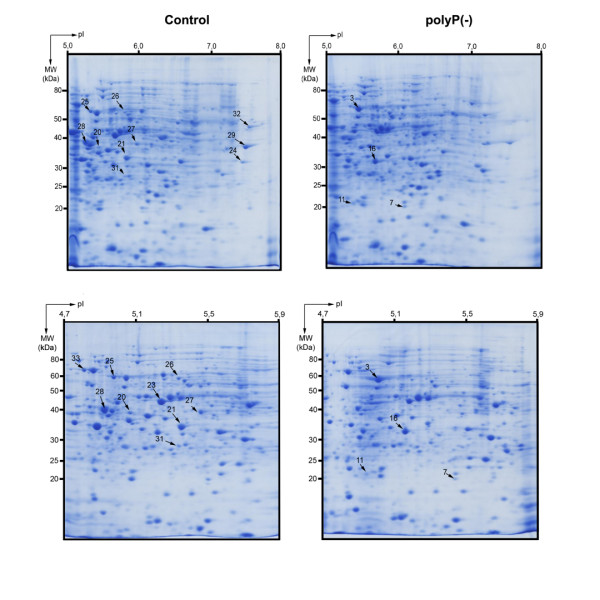
**2D-PAGE gel electrophoresis of polyP-deficient and control *Pseudomonas sp*. B4 cell**. Colonies from *Pseudomonas sp*. B4 polyP-deficient and control cells were grown in LB medium for 48 h. Samples were prepared and analyzed as described in Methods. The upper panels show the separation of proteins in the 5-8 pH range. To have a better resolution of some protein spots a 4.7-5.9 pH range was used (lower panels). Numbers with arrows indicate the spot numbers used for MS/MS analyses (Tables 1 and 2).

**Figure 5:**
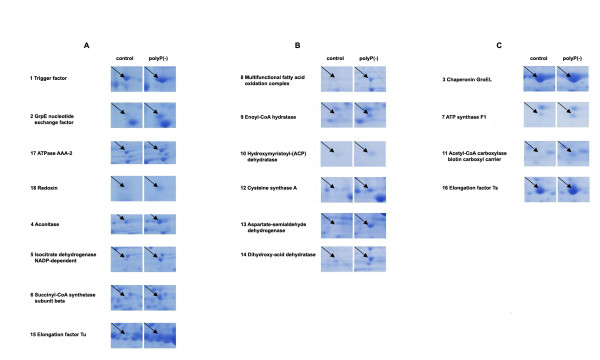
**Summary of protein spots identified whose expression increases during polyP deficiency**. A- Planktonic cultures, exponential phase. B- Planktonic cultures, stationary phase. C- Colonies grown on LB agar plates.

**Figure 6:**
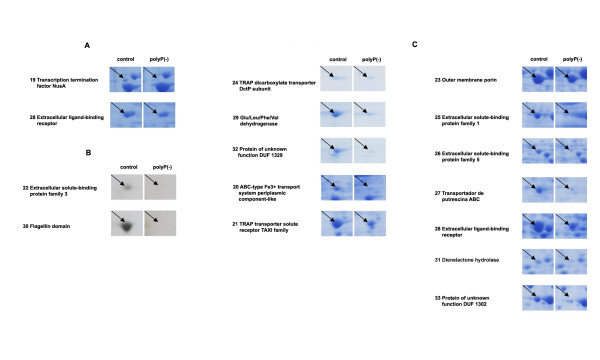
**Summary of protein spots identified whose expression decreases during polyP deficiency**. A, Planktonic cultures from exponential phase. B, Planktonic cultures from stationary phase. C, Colonies grown on LB agar plates.

To gain insight into the effect of polyP deficiency and the metabolic adjustments taking place during the cellular response, the proteomes of *Pseudomonas sp*. B4 polyP-deficient and control cells were compared by two-dimensional gel electrophoresis (2D-PAGE) (Figure 4). We analyzed extracellular and total cell-free proteomes from planctonic cells grown in LB medium during exponential and stationary phase of growth and also analyzed the total cell-free proteome of the colony biofilm. These 8 samples were analyzed by using biological and experimental duplicates. This procedure yielded 81 spots of interest (proteins differentially expressed under polyP-deficiency) that were analysed by mass spectrometry resulting in 78 proteins that could be identified. Thirty-five different proteins whose expression consistently changed between the control and polyP-deficient cells in the conditions assayed are listed in Tables 1 and 2. Gel spots details are seen in Figures 5 and 6. Next, a summary of some relevant functional categories over- and under-represented during polyP deficiency is presented.

**Table 1:** Summary of Gene Ontology categories of overrepresented proteins whose expressions increase during polyP deficiency in *Pseudomonas sp*. B4.

GO Term Annotation	Spot	Protein Name IPR	NCBI Accession	**Theo**. Mr (kDa)/PI	**Exp**. Mr (kDa)/PI	Species/Coverage	Mascot Score
**Biological Process**							

Protein folding GO:0006457	1 e, l	Trigger factor IPR008881	gi: 145575278	48.3/4.78	55/5.1	*Pseudomonas mendocina *ymp/44%	1359
	2 e, l	GrpE nucleotide exchange factor IPR000740	gi: 60549562	20.4/4.9	24/5.1	*Pseudomonas putida*/29%	267
	3 st, a	Chaperonin GroEL IPR012723	gi: 146308703	56.8/5.02	55/5.2	*Pseudomonas mendocina *ymp/35%	674
Tricarboxylic acid cycle GO:0006099	4 e, l	Aconitase IPR004406	gi: 145575802	94.2/5.24	95/5.8	*Pseudomonas mendocina *ymp/32%	1715
	5 e, l	Isocitrate dehydrogenase, NADP-dependent IPR004436	gi: 146307420	82.1/5.63	90/6.3	*Pseudomonas mendocina *ymp/24%	1130
Metabolic process GO:0008152	6 e, l	Succinyl-CoA synthetase IPR005809	gi: 146307523	41.8/5.5	49/6.5	*Pseudomonas mendocina *ymp/34%	654
ATP synthesis proton transport GO:0015986	7 st, a	ATP synthase F1, delta subunit IPR000711	gi: 146309623	19/5.87	20/5.6	*Pseudomonas mendocina *ymp/40%	310
Fatty acid metabolic process GO:0006631	8 st, l	Fatty acid oxidation complex IPR006180	gi: 146306611	77.5/5.58	70/6.5	*Pseudomonas mendocina *ymp/51%	159
Metabolic process GO:0008152	9 st, l	Enoyl-CoA hydratase IPR001753	gi: 146307097	29.8/5.67	27/6.3	*Pseudomonas mendocina *ymp/54%	61
Fatty acid biosynthetic process GO:0006633	10 st, l	Hydroxymyristoyl-(ACP) dehydratase IPR010084	gi: 146308063	16.8/6.3	15/7.5	*Pseudomonas mendocina *ymp/67%	106
	11 st, a	Acetyl-CoA carboxylase biotin carboxyl carrier IPR001249	gi: 26987297	16.2/4.95	20/4.8	*Pseudomonas putida *KT2440/20%	415
Cysteine biosynthetic process serine GO:0006535	12 st, l	Cysteine synthase IPR005859	gi: 146306821	34.4/5.89	37/6.5	*Pseudomonas mendocina *ymp/32%	451
Amino acid biosynthetic process GO:0008652	13 st, l	Aspartate-semialdehyde dehydrogenase IPR012280	gi: 146307742	40.5/5.33	40/6	*Pseudomonas mendocina *ymp/63%	128
Branched chain family amino acid biosynthetic process GO:0009082	14 st, l	Dihydroxy-acid dehydratase IPR004404	gi: 146309219	66.2/5.69	60/6.5	*Pseudomonas mendocina *ymp/44%	114
Translational elongation GO:0006414	15 e, l	Elongation factor Tu IPR004541	gi: 146308925	43.9/5.38	45/5.8	*Pseudomonas mendocina *ymp/43%	847
	16 st, a	Elongation factor Ts IPR001816	gi: 146308073	30.5/5.22	30/5.2	*Pseudomonas mendocina *ymp/52%	895
**Molecular function**							
ATP binding GO:0005524	17 e, l	ATPase AAA-2	gi: 146308654	95/5.32	90/5.9	*Pseudomonas mendocina *ymp/40%	2404
Antioxidant activity GO:0016209	18 e, l	Alkyl hydroperoxide reductase IPR000866	gi: 119860085	17.6/5.02	17/5.1	*Pseudomonas putida *W619/24%	149

GO: Gene Ontology Term Annotation; Spot numbers correspond to spots in 2D-PAGE; Growth Phase (e:exponential; st: stationary); Culture Medium LB (l: liquid; a: agar plate); IPR: InterPro entry; NCBI accession number from NCBI database; Theo. Mr (kDa)/PI: theoretical molecular mass and isoelectric point; Exp. Mr (kDa)/PI, experimental molecular mass and isoelectric point estimated from the 2D-PAGE gels.

**Table 2:** Summary of Gene Ontology categories of overrepresented proteins whose expressions decrease during polyP deficiency in *Pseudomonas sp*. B4.

GO Term Annotation	Spot	Protein Name IPR	NCBI Accession	**Theo**. Mr (kDa)/PI	**Exp**. Mr (kDa)/PI	Species/Coverage	Mascot Score
**Biological Process**							

Regulation of transcription termination GO:0031554	19 e, l	Transcription termination factor NusA IPR010213	gi: 146308624	54.6/4.52	70/5.0	*Pseudomonas mendocina *ymp/16%	508
Transport GO:0006810	20 st, a	ABC-type Fe^3+ ^transport system periplasmic component-like IPR011587	gi: 146306364	38.1/5.27	38/5.3	*Pseudomonas mendocina *ymp/50%	627
	21 st, a	TRAP transporter solute receptor, TAXI family IPR011852	gi: 146309574	33.3/5.74	35/6	*Pseudomonas mendocina *ymp/26%	808
	22 st, l	Extracellular solute-binding protein, family 3 IPR001638	gi: 146309284	27.6/4.79	27/5	*Pseudomonas mendocina *ymp/66%	545
	23 st, a	Outer membrane porin IPR005318	gi: 146309320	46.6/6.03	45/5.2	*Pseudomonas mendocina *ymp/22%	411
	24 st, a	TRAP dicarboxylate transporter, DctP subunit IPR004682	gi: 146307449	37.6/7.04	35/7.5	*Pseudomonas mendocina *ymp/30%	292
	25 st, a	Extracellular solute-binding protein, family 1 IPR006059	gi: 146307075	64.8/4.98	60/5	*Pseudomonas mendocina *ymp/44%	1080
	26 st, a	Extracellular solute-binding protein, family 5 IPR000914	gi: 146305880	59.3/5.72	55/5.3	*Pseudomonas mendocina *ymp/16%	354
Polyamine transport GO:0015846	27 st, a	Transportador de putrescina ABC IPR005893	gi: 70730588	42/6.67	40/5.4	*Pseudomonas fluorescens *Pf-5/14%	122
Transport GO:0006810	28 e/l, st/a	Extracellular ligand-binding receptor IPR001828	gi: 146306419	39.4/5.12	40/5.3	*Pseudomonas mendocina *ymp/20%	585
Amino acid metabolic process GO:0006520	29 st, a	Glu/Leu/Phe/Val dehydrogenase IPR006097	gi: 146307897	37.1/5.85	40/7.5	*Pseudomonas mendocina *ymp/21%	366
Ciliary or flagellar motility GO:0001539	30 st, l	Flagellin domain IPR001492	gi: 146307857	49.9/5.04	50/5	*Pseudomonas mendocina *ymp/6%	280
**Molecular function**							
Hydrolase activity GO:0016787	31 st, a	Dienelactone hydrolase IPR002925	gi: 146307513	27.8/5.45	30/5.3	*Pseudomonas mendocina *ymp/24%	411
Unknown function	32 st, a	Protein of unknown function DUF1329	gi: 146308674	51.4/8.3	50/7.8	*Pseudomonas mendocina *ymp/50%	1200
	33 st, a	Protein of unknown function DUF1302	gi: 77457132	64.1/5.15	65/4.9	*Pseudomonas fluorescens *PfO/13%	340

Conditions and abbreviations are the same as those in Table1.

#### Energy metabolism

The polyP-deficient strain overexpressed three TCA cycle enzymes during exponential phase: aconitase, isocitrate dehydrogenase and succinyl-CoA synthetase. The last two proteins are directly involved in producing NADH and GTP (or ATP) respectively. Additionally, in solid medium, this strain overexpressed ATP synthase F1 (delta subunit) that synthesizes ATP coupled to an electrochemical protons gradient in the respiratory chain [23]. Several catabolic pathways converge on the TCA cycle and particularly; beta-oxidation is the process by which fatty acids are broken down to generate acetyl-CoA, the entry molecule for the TCA cycle. Curiously, during stationary phase of planktonic polyP(-) cultures, cells overexpressed two proteins belonging to the mutifunctional fatty acid oxidation complex that generates acetyl-CoA species: enoyl-CoA hydratase and 3-hydroxyacyl-CoA dehydrogenase. Both enzymes catalyze successive reactions, and their substrates are also related to polyhydroxyalkanoates (PHA) biosynthesis [24]. This polymer is accumulated in anaerobic cultures during stages in which polyPs are degraded [25], and perhaps low polyP levels may enhance PHA accumulation. It would be interesting to find out if the absence of polyP affected other storage biopolymers such as triacylglycerols (TAG), wax esters, polyhydroxyalkanoates (PHA) and glycogen.

#### Protein folding and stress response

Three proteins involved in protein folding were overexpressed during exponential phase by the polyP(-) strain: trigger factor, GrpE and ClpB. Additionally, GroEL was increased in the same strain during stationary phase. All of them are considered chaperones that prevent inappropriate molecular interactions by binding to hydrophobic regions in non-native proteins and allow proper protein folding acting as a molecular network [26]. Trigger factor is a ribosome-associated bacterial chaperone that begins nascent protein folding in an ATP-independent manner [27,28]. On the other hand, GrpE is a co-chaperone that works as a nucleotide exchange factor on a DnaK domain, whereas ClpB rescues stress-damaged proteins from an aggregated state asissted by DnaK [27,29]. GroEL interacts with recently synthesized proteins after their release from the ribosome [26]. With the exception of trigger factor, the other three chaperones form an ATP-dependent network.

Also, an alkyl hydroperoxide reductase (peroxiredoxin) was overexpressed in exponential phase of polyP-deficient cells. This enzyme reduces peroxides to water or alcohols and prevents oxidative stress in bacteria coupled to the TCA cycle and respiratory chain. Additionally, it regenerates the NAD pool and keeps oxidative and reducing balance [30,31]. Peroxiredoxin could act as protection factor against ROS generated by the stress caused by low polyP levels. Finally, increased levels of the translational factors EF-Tu and EF-Ts were found during polyP scarcity. This response has also been described in *E. coli *during acid stress and heavy metal (cobalt) exposure. It is suggested that these elongation factors could fold proteins in a way similar to that of stress chaperones [32]. Finally, as the GTP hydrolysis step is catalysed by EF-Tu, which binds to the large ribosomal subunit, it has been proposed that the interaction between polyphosphate and the large ribosomal subunit promotes translation fidelity by influencing the EF-Tu GTPase reaction [33].

Altogether, these results suggest that during polyP scarcity a general stress state occurred and cells succeeded by overexpressing protein-folding chaperones.

#### Transport proteins

From the 17 total proteins identified whose expressions decreased during lack of polyP, 10 were identified as transporters. Energy consuming ABC-type transporters responsible for carrying different solutes such as sugars, peptides, polyamines, amino acids and Fe^3+ ^were identified. Also, C4-dicarboxylates TRAP transporters and outer membrane protein OprE, which has been involved in virulence process in the genus *Pseudomonas *[34], were reduced in polyP(-) cells.

#### Other processes and hypothetical proteins

The present study also yielded some results that appear to be conflicting. We, and others, have demonstrated that despite the lack of motility of polyP-deficient cells, the flagellum was intact (as seen by using transmission electron microscopy). Nevertheless, we found flagellin, the major component of flagella filaments, diminished in the total and extracelullar proteome of polyP-deficient cells.

Finally two protein spots present in the total proteome matched ORF sequences designated 'hypothetical' or 'conserved hypothetical" proteins. These hypothetical proteins identified here should be subjected to further characterization to confirm their possible role in polyP metabolism and to ascertain their true biological function.

## Discussion and Conclusions

PolyP has numerous and diverse biological functions that have been discovered mainly by studying ppk1 mutants in bacteria. A *P. aeruginosa PAO1 ppk1 *null mutant exhibits pleiotropic phenotypes including decreased virulence, defective in motility, quorum sensing, biofilm formation and failure in responses to various stresses [13,15,22]. Many of these features were also observed in *ppk1 *mutants of other bacteria such as *Vibrio cholerae, Salmonella, Shigella *and others [35,36]. Nevertheless, new functional and structural defects were found in our recombinant cells that overexpressed the exopolyphosphatase, the enzyme in charge of polyP degradation to Pi. These results might be explained by the higher extent of polyP depletion when using this approach. In the genus *Pseudomonas*, despite the lack of detectable PPK1 activity (<1% of wild type), these mutants still possess as much as 20% of the wild-type levels of poly P as is the case of *P. aeruginosa *PAO1 [22]. We previously reported that the overexpression of exopolyphosphatase removed more than 95% of cellular polyP [21].

The changes observed in the colony morphology are not surprising taking into account that polyP deficient *P. aeruginosa *PAO1 cells fails to produce extracellular polysaccharide [22]. Similar results and an additional change in the LPS profile were seen in our polyP-deficient cells. Although, the LPS structure of *Pseudomonas sp*. B4 is not known in detail it can be speculated that the change seen in the LPS could be due to an alteration in the phosphate moiety of the LPS core or that polyP regulates some enzyme able to modify the LPS. Further experiments should be done to clarify this finding but it will be interesting to find out if some of the LPS kinases reported in the genus *Pseudomonas *(such as WaaP [37]) could use polyP instead of ATP during phosphorylation of Heptose I in the inner core of LPS. Furthermore, taking into account the role of LPS during pathogenesis development in many bacteria, this change might explain some dysfunction during virulence of polyP-deficient bacteria.

Bacterial cell division occurs through the formation of an FtsZ ring (Z ring) at the site of division. The ring is composed of the tubulin-like FtsZ protein that has GTPase activity and the ability to polymerize in vitro (reviewed in [38]). Our observation of cell division failure in polyP-deficient cells during entry into the stationary phase is in agreement with the finding that during polyP-deficiency energy metabolism, and particularly nucleoside triphosphate (NTP) formation, was affected (see below). As seen in Figure 3, the cells were apparently able to form the septum, but did not complete the separation process. It is possible that polyP scarcity affects the function of FtsZ, since its GTPase activity needs both, GTP and a bivalent ion. Considering that polyP can provide both, phosphate for the generation of GTP ([16,17] and bivalent metals [35], the absence of this biopolymer could block indirectly the polymerisation of Z ring, which would explain the observed phenotype. Curiously, the enzyme in charge of GTP synthesis from polyP in *P. aeruginosa *(PPK2), was induced 100-times in the stationary phase [16]. In this phase of growth GTP is necessary for the synthesis of alginate and other functions such as cellular division. At present, we cannot discard that other proteins from the divisome, that also employ GTP for their activity, are affected by the absence of polyP.

**Figure 7:**
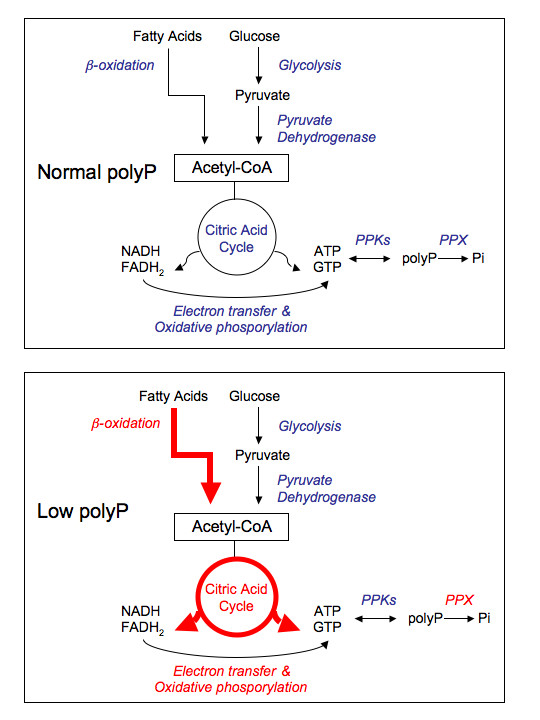
**Working model proposed for the metabolic adjustment of bacterial cells during polyP deficiency**. In red, metabolic pathways in which several of its components are overexpressed during polyP scarcity.

Living cells constantly require energy for maintaining their highly organized structures, synthesize cellular components, generate electric currents, and many other processes. PolyP acts as a reserve for high energy Pi and regulates intracellular ATP in combination with oxidative and substrate level phosphorylation. Our proteomic data support the hypothesis that polyP is an important component for energy regulation, and particularly in ATP regeneration [39]. During polyP deficiency, cells would prevail by increasing the flux of important energy generating pathways such as β-oxidation, citric acid cycle and oxidative phosphorylation as proposed in Figure 7. We found eight different proteins related to these pathways increased during polyP deficiency and in the case of the TCA cycle enzymes two of them are directly involved in the generating NADH and GTP by their activity (see Table 1). Interestingly, a previous link between polyP and the TCA cycle was reported in *P. aeruginosa*. AlgR2, a global transcriptional factor, positively regulates nucleoside diphosphate kinase (Ndk) and succinyl-CoA synthetase, enzymes critical in nucleoside triphosphate (NTP) formation [40]. Thus, AlgR2 positively regulates the production of alginate, GTP, ppGpp and inorganic polyP in *P. aeruginosa *[41]. It is possible then that polyP-deficiency induces AlgR2 expression to increase GTP and polyP production. This could explain the increase of succinyl-CoA synthetase in our polyP deficient cells.

Active transport of ion and molecules across the membrane consumes energy and ATP. We found that the majority of protein spots decreasing their levels in polyP(-) cells belong to the transport protein category (see Table 2). It is possible that diminishing energy-consuming processes such as active transport can help the cells to overcome this polyP deficiency.

The defects in the *ppk1 *mutant described in *P. aeruginosa *[22,42], and those seen in the same *E. coli *mutant [10], suggest a failure to respond to a variety of stresses. We found that the levels of many important chaperones and enzymes related to stress response are increased in polyP deficient cells. It is suggested that a general stress response occurs during polyP deficiency and cells prevail by augmenting the levels of general chaperones and enzymes that would remove reactive oxygen species. In fact, our previous results showed that growth of *Pseudomonas sp*. B4 in certain conditions generates an oxidative stress and produced a massive increase of polyP [43].

Altogether the results presented in this communication demonstrate the usefulness of proteomics to study the effect of polyP deficiency in order to generate new hypothesis to clarify its role in bacteria. New suggestions such as the possible link between the central metabolic pathways and polyP metabolism proposed here should be the focus of future metabolic flux experiments. The integrations of OMICS technologies will definitely help in elucidating the role of polyP in bacteria and its importance during pathogenic process.

In summary, polyP has numerous and varied biological functions in bacteria that have been discovered mainly by studying its deficiency. To better understand the function of polyP we used broad-host-range constitutive and regulated vectors to deplete cellular polyP and found new functional and structural changes. In addition, it is generally accepted that energy supply of the cells could be severely compromised in the absence of polyP. We confirmed this evidence by using differential proteomic studies and suggested that during polyP scarcity energy metabolism and particularly nucleoside triphosphate (NTP) formation were affected, generating a general stress condition. We propose that bacterial cells prevail by increasing the flux of energy-generating metabolic pathways such as tricarboxilic acid (TCA) cycle and β-oxidation and by reducing energy-consuming ones such as active transporters and amino acid biosynthesis.

## Methods

### Bacterial strains and growth conditions

*Pseudomonas *sp. B4 wt, control (pMLS7) and polyP-deficient (pS7PPX1) recombinant strains were previously obtained [21] and grown aerobically at 37°C on Luria-Bertani (LB) rich medium supplemented with trimetropim (50 μg/ml). When required, LB plates (1,5% (w/v) of Bacto-agar) were used for obtaining cells from the colonies after 48 h of growth.

### Optical and Electron microscopy

Unstained cells from the different cultures were routinely examined for the presence of polyP granules by transmission electron microscopy [43]. Cells were mixed and dispersed in distilled water and added onto carbon-coated nickel grids. The drops containing the microorganisms were drained off with filter paper and air dried during 30-50 s. Electron microscopy was performed with a Philips Tecnai 12 electron microscope using 80 kV accelerating voltage (Electron Microscopy Laboratory, Pontificia Universidad Católica de Chile). Optical microscopy was routinely performed in an Olympus BX50 microscope (Olympus Corporation, Japan).

### LPS analysis

Culture samples were adjusted to an optical density at 600 nm of 2.0 in a final volume of 100 μl. Then, proteinase K-digested whole-cell lysates were prepared as described previously [44], and LPS was separated on 14% acrylamide gels using a Tricine-sodium dodecyl sulfate (SDS) buffer system [45]. Gel loadings were normalized so that each sample represented the same number of cells. Gels were silver stained by a modification of the procedure of Tsai and Frasch [46,47].

### Samples preparation and 2D-PAGE

Cells (200 mg) were harvested by centrifugatio n (7,000 × g for 15 min at 25°C) from liquid cultures or were collected with an inoculation loop from agar plates. Pellets were washed four times in sonication buffer (40 mM Tris pH 8.15; 1 mM PMSF). Cells were disrupted by sonication (Misonix XL2020) and the sample was incubated on ice for 10 min with 50 μg/ml of DNAase. Cell debris was removed by centrifugation and then the sample was washed and concentrated (to half of the total volume) by using a Microcon^® ^YM-3 filter unit (10,000 × g, 4°C). Protein quantitation was performed using Bio-Rad Protein Assay^® ^system. A total of 500 μg of proteins was precipitated through Ready-Prep 2D Cleanup Bio-Rad^® ^kit.

Precipitated proteins were resuspended in 300 μL IEF buffer (7 M urea, 2 M thiourea, 4% CHAPS, 0.0002% bromophenol blue) followed by the addition of DTT to 100 mM and 0.2% Bio-Rad ampholytes and the sample mix was incubated for 1 h at 25°C. The entire volume was loaded in the Protean^® ^IEF focusing tray (17 cm) using the following strips pH ranges: 4.7-5.9/5-8/3-10NL (ReadyStrip™ IPG) that were actively rehydrated at 50 V for 12 h. The focusing step was performed at 250 V for 15 min; 2,000 V for 2 h; 8,000 V for 4 h and finally 10,000 V for 11 h, all the steps at 20°C. Focused proteins in the strip were then incubated at 25°C with gentle agitation for 15 min in equilibrium buffer (6 M urea; 2% SDS; 0,05 M Tris/Cl pH 8.8; 20% glycerol) containing 2% DTT and then 15 min in equilibrium buffer containing 2.5% iodoacetamide. Finally, the strip was placed onto a 12.5% polyacrilamide gel for the second dimension in Protean^® ^II (Bio-Rad) system at 50 V for 23 h. The gels were fixed for 1 h (50% ethanol; 2% phosphoric acid), stained for 3 h (0,12% CBB G-250; 10% phosphoric acid; 10% ammonium sulphate; 20% methanol) and then washed three times with 15% methanol.

Digital images of the gels were analyzed and spots quantified using Delta2D v.3.6 software. Spot volume was normalized as a percentage of the total volume of all spots on the corresponding gel and also manually confirmed. The threshold for accepting a meaningful variation was a factor of 2.0 (p < 0,05). A total of 81 proteins spots showing differences in the expression pattern between control and polyP(-) strains (three independent replicates) were selected for further MS analysis.

### In-gel protein digestion and sample preparation

Spots of interest from Coomassie blue-stained 2D gels were excised manually, deposited in 96-well plates and processed automatically in a Proteineer DP (Bruker Daltonics, Bremen, Germany). The digestion protocol used was based on Schevchenko *et al*. [48] with minor variations: gel plugs were washed firstly with 50 mM ammonium bicarbonate and secondly with acetonitrile (ACN) prior to reduction with 10 mM DTT in 25 mM ammonium bicarbonate solution, and alkylation was carried out with 55 mM IAA in 50 mM ammonium bicarbonate solution. Gel pieces were then rinsed with 50 mM ammonium bicarbonate and with ACN, and then dried under a stream of nitrogen. Modified porcine trypsin (sequencing grade; Promega, Madison WI) was added at a final concentration of 16 ng/μl in 25% ACN/50 mM ammonium bicarbonate solution and the digestion took place at 37°C for 6 h. The reaction was stopped by adding 0.5% TFA for peptide extraction. Tryptic peptides were dried by speed-vacuum centrifugation and resuspended in 4 μl of MALDI solution. A 0.8 μl aliquot of each peptide mixture was deposited onto a 386-well OptiTOF™ Plate (Applied Biosystems, Framingham, MA, USA) and allowed to dry at room temperature. A 0.8 μl aliquot of matrix solution (3 mg/mL CHCA in MALDI solution) was then added onto dried digest and allowed to dry at room temperature.

### MALDI peptide mass fingerprinting, MS/MS analysis and database searching

For MALDI-TOF/TOF analysis, samples were automatically acquired in an ABi 4800 MALDI TOF/TOF mass spectrometer (Applied Biosystems, Framingham, MA, USA) in positive ion reflector mode (ion acceleration voltage was 25 kV for MS acquisition and 1 kV for MSMS) and the spectra were stored into the ABi 4000 Series Explorer Spot Set Manager. PMF and MSMS fragment ion spectra were smoothed and corrected to zero baseline using routines embedded in ABi 4000 Series Explorer Software v3.6. Each PMF spectrum was internally calibrated with the mass signals of trypsin autolysis ions to reach a typical mass measurement accuracy of <25 ppm. Known trypsin and keratin mass signals, as well as potential sodium and potassium adducts (+21 Da and +39 Da) were removed from the peak list. To submit the combined PMF and MS/MS data to MASCOT software v.2.1 (Matrix Science, London, UK), GPS Explorer v4.9 was used, searching in the non-redundant NCBI protein database.

### LC-ESI MS/MS analysis

In some specific cases, alternative proteomic techniques were employed to confirm and improve protein identifications. For this purpose, we made use of liquid chromatography coupled to electrospray ion-trap mass spectrometry tandem MS (LC ESI-MS/MS). This was done using an Ultimate 3000 nano LC (Dionex, Amsterdam, The Netherland) and a 75 micrometer I.D, 100 mm reversed-phase column, at a 300 nL/min flow, coupled to a Bruker HCT Ultra ion-trap mass spectrometer (Bruker Daltonics, Bremen, Germany) working in dynamic exclusion mode.

### Database Search

For protein identification, LC ESI MS/MS spectra were transferred to BioTools 2.0 interface (Bruker Daltonics) to search in the NCBInr database using a licensed version of Mascot v.2.2.04 search engine (http://www.matrixscience.com; Matrix Science, London, UK). Search parameters were set as follows: carbamidomethyl cystein as fixed modification by the treatment with iodoacetamide, oxidized methionines as variable modification, peptide mass tolerance of 0.5 Da for the parental mass and fragment masses and 1 missed cleavage site. In all protein identifications, the probability Mowse scores were greater than the minimum score fixed as significant with a p-value minor than 0.05. Selected proteins were based on that who exhibited higher Mascot score and sequence coverage. A total of thirty-three different proteins showing differential expression pattern between polyP+ and polyP- strains (three independent replicates) were selected. Furthermore, theoretical isoelectric points and molecular masses were compared to experimental values of thirty-three proteins were grouped by gene ontology (GO) categories [49].

## Authors' contributions

FPC y CAJ conceived and designed the study; FPC performed some experiments and wrote the manuscript. CV performed proteomic experiments. CM carried out cellular experiments. AP y JPA carried out MS/MS protein identification. CAJ participated in coordination and critical evaluation of the manuscript. All authors read and approved the final manuscript.
